# The trends in lung cancer prevalence, incidence, and survival in Hong Kong over the past two decades (2002–2021): a population-based study

**DOI:** 10.1016/j.lanwpc.2024.101030

**Published:** 2024-02-16

**Authors:** Philip CM. Au, Anne WM. Lee, Victor HF. Lee, Ian CK. Wong, Rina YM. Hui, Ching-Lung Cheung

**Affiliations:** a Department of Pharmacology and Pharmacy, Li Ka Shing Faculty of Medicine, The University of Hong Kong, L02-56, 2/F, Laboratory Block, 21 Sassoon Road, Pokfulam, Hong Kong SAR, China; b Department of Clinical Oncology, Li Ka Shing Faculty of Medicine, The University of Hong Kong, Hong Kong SAR, China; c Laboratory of Data Discovery for Health (D24H), Hong Kong Science Park, Pak Shek Kok, Hong Kong SAR, China; d Research Department of Practice and Policy, School of Pharmacy, University College London, London, UK; e Aston Pharmacy School, College of Health and Life Sciences, Aston University, Birmingham, UK; f Centre of Cancer Medicine, School of Clinical Medicine, Li Ka Shing Faculty of Medicine, The University of Hong Kong, Hong Kong SAR, China

**Keywords:** Lung cancer, Epidemiology, Trends, Prevalence, Incidence, Survival

## Abstract

**Background:**

Over the past decades, significant progress in lung cancer management has been made. However, the trends in prevalence and survival of lung cancer in the Chinese population over the last decade remain unexplored. This study utilised a territory-wide electronic medical database in Hong Kong to provide the most up-to-date and comprehensive analysis of the trends in prevalence, incidence, and survival over the past two decades.

**Methods:**

Descriptive epidemiology study using a retrospective cohort of lung cancer patients from the Clinical Data Analysis and Reporting System (CDARS). 10-year limited-duration prevalence, incidence, and relative period survival were calculated between 2002 and 2021. Sub-groups of age, sex, and comorbidity were examined. The annual percent change (APC) and average annual percent change (AAPC) were estimated using joinpoint regression.

**Findings:**

This study included 87,259 incident cases between 2002 and 2021. The 10-year limited duration prevalence (per 100,000 persons) of lung cancer increased from 153.4 to 228.7 (AAPC: 3.08%). Crude incidence (per 100,000 persons) increased from 55.0 to 70.3 (APC: 1.23%), while age-standardised incidence decreased from 42.9 to 33.2 (APC: −1.32%). The 1-year and 5-year relative period survivals showed an increasing trend but remained low. Disparity in trends was observed among different sex and age groups.

**Interpretation:**

Lung cancer burden has been increasing partly due to population ageing. Although survival showed improvement over the years, it remained low, highlighting the potential need for interventions. Further study exploring the disparity in sex-specific trends is warranted.

**Funding:**

The 10.13039/501100003452Innovation and Technology Commission, Hong Kong.

**Research in context**

**Evidence before this study**

A literature search was performed in PubMed on 31/10/2023 for studies published between 01/01/2000 and 31/10/2023 using the following search string: (((“Lung Cancer”) AND ((((“Prevalence”) OR “Incidence”) OR “Survival")) AND (((“China”) OR (“Taiwan")) OR (“Hong Kong")) AND (“Trend")) OR ((((“Lung Neoplasms" [Mesh]) OR “Lung Neoplasms/epidemiology" [Mesh]) AND ((((“Prevalence" [Mesh]) OR “Incidence" [Mesh]) OR “Mortality" [Mesh]) OR “Epidemiology/trends" [Mesh])) AND (((“China”) OR (“Taiwan")) OR (“Hong Kong"))).

The trends in lung cancer incidence have been examined in various Chinese populations, with the latest study reporting trends up to the year 2019. However, there has been a lack of population-based studies examining the trends in lung cancer prevalence and survival over the last decade in the Chinese populations.

**Added value of this study**

The Clinical Data Analysis and Reporting System (CDARS) database contains longitudinal electronic medical records from the public healthcare sector in Hong Kong. This study utilised a highly representative lung cancer population of 87,259 incident cases between 2002 and 2021 from the CDARS to provide the most up-to-date and comprehensive analysis of the trends in lung cancer incidence, prevalence, and survival over the past two decades in a Chinese population.

**Implications of all the available evidence**

The prevalence of lung cancer patients and survivors continued to rise due to the ageing population and improved survival. The higher comorbidity associated with cancer survivors would lead to higher annual medical expenditures and annual lost productivity. Adaptation and adjustment to disease management and resource allocation would be warranted to tackle the increasing burden of disease on society.

On the other hand, despite continuous improvement in lung cancer survival over the years, the survival remained extremely low. Many countries currently do not have public lung cancer screening programmes. The implementation of lung cancer screening programmes could potentially improve further the survival of lung cancer patients.

Disparity in lung cancer incidence and survival trends existed between females and males. Differences in smoking habits could be one of the reasons. Further study is needed to examine how lifestyle and genetic differences could explain the disparity.

## Introduction

Lung cancer has been one of the most common cancers and the leading cause of cancer deaths in both females and males worldwide.^1^ In 2020, lung cancer accounted for 15.9% of all cancer incidence and 26.4% of all cancer deaths in Hong Kong, with a crude incidence of 72.5 per 100,000 persons and a crude mortality of 52.3 per 100,000 persons.^2^ The Hong Kong Cancer Registry (HKCR) collects information on all cancer patients from both public and private sectors in Hong Kong and publishes summary statistics on cancer incidence and mortality. According to the HKCR, up until 2020, there has been a continuous increase in crude incidence and a continuous decrease in age-standardised incidence over the past 2 decades.^2^ However, one limitation of the HKCR is the lack of longitudinal data and thus reports on the trends in prevalence and survival are not available. In fact, although the recent trends in lung cancer incidence have been examined in various Chinese populations, there has been a lack of population-based studies (from China, Hong Kong, or Taiwan) examining the trends in lung cancer prevalence and survival over the last decade in the Chinese populations. Due to the changing treatment landscape and management of lung cancer, it is important to examine the trends in lung cancer prevalence and survival over the years, as well as among groups with different age, sex, and comorbidity.

Advances in lung cancer management have been a worldwide priority. Over the past two decades, from policies to raise public awareness of lung cancer prevention, to the use of advanced radiology techniques and targeted/immuno-therapies, significant progress in lung cancer management has been made with the aim to reduce disease burden and improve disease survival. The improved survival of lung cancer patients on the other hand could result in an increased use of healthcare resources due to increased prevalence of cancer patients and survivors. Understanding the changing prevalence and survival of lung cancer over these years would help inform future clinical care and public health initiatives. In the current study, we aimed to utilise a territory-wide electronic medical database to provide the most up-to-date and comprehensive analysis of the trends in lung cancer prevalence, incidence, and survival over the past two decades in Hong Kong.

## Methods

### Data source

The Clinical Data Analysis and Reporting System (CDARS) is a territory-wide representative electronic medical database from the Hospital Authority (HA) of Hong Kong. The HA manages forty-three public hospitals and one hundred twenty-two public outpatient general and specialist clinics in Hong Kong.^3^ Approximately 90% of lung cancer patients in Hong Kong are under the HA’s care.^4^ The CDARS has an ethnically homogeneous population of about 92% Han Chinese.^5^ It contains clinical records from outpatient, emergency, and inpatient, including diagnosis, dispensing, clinical procedures and operations, laboratory tests, and death registry records.

### Study population

The main cohort consisted of all patients first diagnosed with lung cancer between 2002 and 2021 (20 years) in the CDARS database. Lung cancer was defined as an ICD-9-CM diagnosis code of 162.x.^6^ Eligible patients’ demographic information and diagnosis records since 1993 were retrieved. No patient had missing date of birth and one patient diagnosed in 2002 was excluded due to missing date of death. Missing date of death referred to those recorded as deceased, but with no recorded date. For the calculation of 10-year limited-duration prevalence between 2008 and 2021, additional demographic data from lung cancer patients diagnosed between 1999 and 2001 were retrieved to supplement the main cohort. Patients’ sex was defined as the sex assigned at birth.

### Statistical analysis

#### Prevalence calculation

Since the “end” date of lung cancer could not be ascertained, the limited-duration prevalence of lung cancer was estimated. It was calculated as the proportion of people alive in a certain year who had lung cancer diagnosed within a pre-defined time period.^7^ A 10-year limited-duration prevalence was calculated yearly between 2008 and 2021. A prevalent case in a certain year was defined as a lung cancer patient who was first diagnosed within a 10-year period prior and was still alive at the start of that year. The prevalence was defined as: *The total number of prevalent cases in a year/the mid-year population in that year.* The mid-year population was retrieved from the statistics reported by the Hong Kong Census and Statistics Department.^8^ Under the limited-duration approach, the definition of prevalent cases varies according to the time period chosen. A longer period would include more long-term survivors of the disease, while a shorter period would limit the prevalent cases to those with more current diagnosis. Therefore, sensitivity analysis which limited the prevalent cases to patients with current lung cancer was also performed using the 5- and 3-year limitation-duration prevalence.

#### Incidence calculation

Both crude and age-standardised incidence were calculated yearly between 2002 and 2021. Incidence was defined as: *The total number of incident cases in a year/the mid-year population in that year.* An incident case in a certain year was defined as a lung cancer patient who was first diagnosed in that year without any lung cancer records within 10 years before the diagnosis. The mid-year population was retrieved from the statistics reported by the Hong Kong Census and Statistics Department.^8^ The 2015 world population from the World Bank was used as the standard population.^9^ A direct standardisation method was adopted.

#### Survival calculation

Relative period survival was calculated in 3-year intervals between 2004 and 2021. Traditional survival calculation starts from a defined cohort at the index year and follows forward. Therefore, there is always a certain lag in years in survival estimates, depending on the length of the follow-up. Period survival on the other hand is a method which does not utilise a forward follow-up in estimating patients’ survival.^10^ Period survival calculation utilises the past survival information of patients who survived up to the index year (or year period).^10^ Therefore, this method removes the need for a long forward follow-up window and allows more up-to-date survival estimates, which is a particularly useful way to examine the most recent survival trends. A 3-year period was opted in this study to preserve sufficient survival data for survival estimation. Relative survival on the other hand takes into account the deaths due to other causes (*i.e.* the expected survival of the general population).^11^ This allows fair cancer survival comparisons between years even when the expected survival of the general population varies in different years. The expected survival was estimated by the Ederer II method^12^ using life tables from the Hong Kong Census and Statistics Department.^13^ For age standardisation, the world lung cancer population from GLOBOCAN 2020 was used as the standard population.^14^ To account for sparse age-specific data, age standardisation was computed according to the Brenner approach.^15^ Both 1-year and 5-year relative period survival was calculated using *R (version 4.2.0)* package “*periodR*”.^16^

#### Age, sex and comorbidity sub-groups

Age and sex are two key demographic characteristics in defining sub-groups with different disease risks. The comorbidity profiles, genetic compositions, and environmental determinants among people of different age and sex vary widely. Therefore, age- and sex-specific prevalence, incidence and survival were calculated to examine the differences in trends. For the age sub-groups, lung cancer patients were divided into four age groups: below 40, 40 to 59, 60 to 79, and above 79. For incidence and survival analyses, the age of the patients was defined as the age at lung cancer diagnosis. For prevalence analysis, the age of the surviving patients was defined as the age at the mid-year of the respective index year. In addition, for survival analysis, lung cancer patients were also divided into comorbidity sub-groups according to the Charlson Comorbidity Index (CCI) score proposed by *Quan* et al. *2005*.^17^ Worse comorbidity profiles have been shown to associate with worse lung cancer survival.^18^ Therefore, the survival trends in 3 sub-groups of comorbidity severity were also examined: Severe comorbidity was defined as a CCI score of 3 or above; mild comorbidity was defined as a CCI score between 1 and 2; no comorbidity was defined as a CCI score of 0. The ICD-9-CM diagnosis records in the 5 years before lung cancer diagnosis were used to calculate the CCI scores with weights published by *Quan* et al. *2005*.^17^ Lung cancer (ICD-9-CM 162.x) was not counted as a baseline comorbidity. Detailed ICD-9-CM definition of each comorbidity was listed in Supplementary Table S1.

#### Trend analysis

The annual percent change (APC) and average annual percent change (AAPC) were estimated using joinpoint regression.^19^ The best model was selected based on the weighted Bayesian Information Criterion (BIC). For models with no joinpoint, only APCs were reported. For the period survival trends, since survival was estimated every three years, the total number of estimates was thirded. Therefore, no joinpoint was assumed in the regression analyses and only APCs were reported. All joinpoint analyses and plots were generated using the software *Joinpoint Regression Program (5.0.2)* by the U.S. National Cancer Institute.^20^

### Comparison with the HKCR

To assess the representativeness of the study cohort to the lung cancer population in Hong Kong, the age-standardised incidence reported in the present study was compared to the age-standardised incidence reported by the HKCR.^2^ Incidence trends were plotted using the annual sex-specific age-standardised incidence estimates and AAPCs from the cancer statistics reports.^2^ The HKCR uses the Segi (1960) standard population.^21^ For comparison, the sex-specific incidence in the present study was age-standardised to the Segi (1960)^21^ and the World Bank (2015)^9^ standard populations, separately.

### Ethics approval

The study was approved by the Kowloon West Cluster Research Ethics Committee (Ref: KW/EX-22-082 (177-02)); the Joint Chinese University of Hong Kong–New Territories East Cluster Clinical Research Ethics Committee (Ref: 2020.472); the Hong Kong East Cluster Research Ethics Committee (Ref: HKECREC-2020-058); the Kowloon Central Cluster/Kowloon East Cluster Research Ethics Committee (Ref: KC/KE-20-0188/ER-4 & KC/KE-20-0324/ER-4); the Institutional Review Board of the University of Hong Kong/Hospital Authority Hong Kong West Cluster (Ref: UW 20–384); and the New Territories West Cluster Research Ethics Committee (Ref: NTWC/REC/20086).

### Role of the funding source

The study was funded by the Innovation and Technology Commission (ITC)–Hong Kong under the Partnership Research Programme scheme (PRP/067/20FX). The funders had no role in the study design, data collection, data analysis, interpretation, writing of the report, and the decision to submit the paper for publication.

## Results

### Trends in lung cancer 10-year limited duration prevalence

**Fig. 1:**
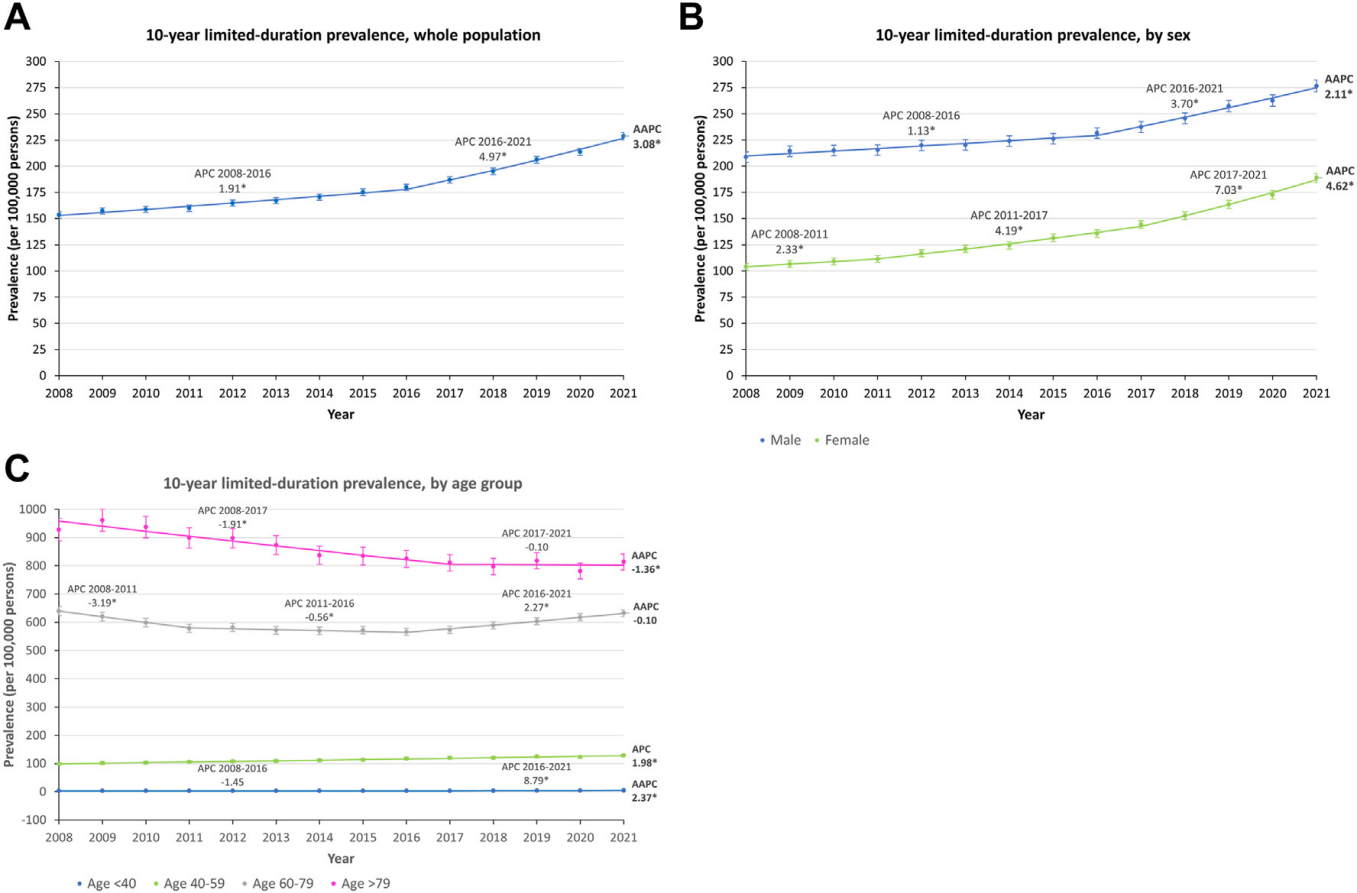
**The trends in lung cancer 10-year limited-duration prevalence between 2008 and 2021.** (A) Whole population; (B) by sex; (C) by age group. AAPC: average annual percent change, APC: annual percent change. ∗Indicates significant (p ≥ 0.05) APC/AAPC.

Between 2008 and 2021, the number of prevalent lung cancer cases each year increased from 10,671 to 16,952 (Table 1). Around 60% of the prevalent cases were male, and around 80% of the prevalent cases were aged 60 or above (Table 1). Over the years, the percentage of males gradually decreased, from 64.3% in 2008 to 55.1% in 2021, whereas the percentage of different age groups remained relatively stable (Table 1). The overall prevalence (per 100,000 persons) increased from 153.4 to 228.7, with an AAPC of 3.08% (95% CI: 2.85 to 3.31; p < 0.001) (Fig. 1A) (Supplementary Table S2). Joinpoint analysis estimated a change in trend in the year 2016 where the APC increased from 1.91% (95% CI: 1.63 to 2.20; p < 0.001) to 4.97% (95% CI: 4.45 to 5.49; p < 0.001) (Fig. 1A) (Supplementary Table S3). Similar increasing trends were observed in sensitivity analysis using 5- and 3-year limited duration prevalences (Supplementary Fig. S1).

**Table 1:** Age and sex distribution of prevalent lung cancer cases between 2008 and 2021.

Year	Total number of prevalent cases	By sex		By age group			
		Male (%)	Female (%)	Age < 40 (%)	Age 40–59 (%)	Age 60–79 (%)	Age > 79 (%)
2008	10,671	6864 (64.3)	3807 (35.7)	126 (1.2)	2299 (21.5)	6129 (57.4)	2117 (19.8)
2009	10,970	7037 (64.1)	3933 (35.9)	117 (1.1)	2383 (21.7)	6163 (56.2)	2307 (21)
2010	11,143	7077 (63.5)	4066 (36.5)	108 (1)	2431 (21.8)	6218 (55.8)	2386 (21.4)
2011	11,308	7112 (62.9)	4196 (37.1)	112 (1)	2512 (22.2)	6244 (55.2)	2440 (21.6)
2012	11,779	7311 (62.1)	4468 (37.9)	99 (0.8)	2586 (22)	6530 (55.4)	2564 (21.8)
2013	11,993	7332 (61.1)	4661 (38.9)	108 (0.9)	2618 (21.8)	6634 (55.3)	2633 (22)
2014	12,324	7488 (60.8)	4836 (39.2)	114 (0.9)	2684 (21.8)	6875 (55.8)	2651 (21.5)
2015	12,779	7612 (59.6)	5167 (40.4)	106 (0.8)	2725 (21.3)	7208 (56.4)	2740 (21.4)
2016	13,189	7812 (59.2)	5377 (40.8)	97 (0.7)	2823 (21.4)	7462 (56.6)	2807 (21.3)
2017	13,821	8052 (58.3)	5769 (41.7)	109 (0.8)	2893 (20.9)	7946 (57.5)	2873 (20.8)
2018	14,543	8378 (57.6)	6165 (42.4)	120 (0.8)	2890 (19.9)	8603 (59.2)	2930 (20.1)
2019	15,477	8811 (56.9)	6666 (43.1)	126 (0.8)	2995 (19.4)	9266 (59.9)	3090 (20)
2020	15,980	8968 (56.1)	7012 (43.9)	143 (0.9)	2936 (18.4)	9869 (61.8)	3032 (19)
2021	16,952	9346 (55.1)	7606 (44.9)	134 (0.8)	3046 (18)	10,588 (62.5)	3184 (18.8)

Increasing trends in prevalence (per 100,000 persons) were observed for both sexes, of which males (AAPC: 2.11%; 95% CI: 1.90 to 2.31; p < 0.001) showed a smaller percentage increase than females (AAPC: 4.62%; 95% CI: 4.13 to 5.11; p < 0.001) (Fig. 1B). However, lung cancer prevalence was constantly higher in males across the years, ranging from 208.6 to 276.3, compared to the range from 103.8 to 188.7 in females (Fig. 1B) (Supplementary Table S2). For both sexes, joinpoint analysis estimated an upward shift in trend around the years 2016–2017 (Fig. 1B) (Supplementary Table S3). Similar increasing trends in males and females were observed in sensitivity analysis using 5- and 3-year limited duration prevalences (Supplementary Fig. S1).

The trends in lung cancer prevalence (per 100,000 persons) among different age groups were mixed (Fig. 1C). The population aged above 79 showed a decreasing trend with an AAPC of −1.36% (95% CI: −1.97 to −0.75; p < 0.001), while the population aged between 60 and 79 showed an overall stable trend (p for AAPC = 0.413) (Supplementary Table S3). When examining individual joinpoint segments, both age groups showed an upward shift in trend after 2016–2017 (Fig. 1C) (Supplementary Table S3). Among different age groups, the highest lung cancer prevalence was observed in older age groups, with prevalence ranged between 813.5 and 928.1 for those aged above 79 and between 566.1 and 639.6 for those aged between 60 and 79 (Fig. 1C) (Supplementary Table S2). For younger populations, although there was an overall increasing trend, the prevalence remained low (Fig. 1C) (Supplementary Table S2). Nonetheless, the increasing trends observed in the whole population attenuated in all age groups. Sensitivity analysis using 5- and 3-year limited duration prevalences showed comparable trends among different age groups (Supplementary Fig. S1).

### Trends in lung cancer incidence

**Fig. 2:**
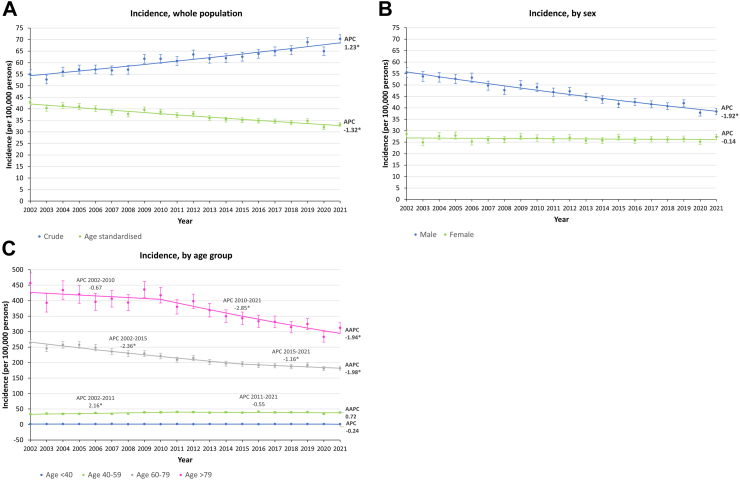
**The trends in lung cancer incidence between 2002 and 2021.** (A) Whole population; (B) by sex, age-standardised; (C) by age group. AAPC: average annual percent change, APC: annual percent change. ∗Indicates significant (p ≥ 0.05) APC/AAPC.

Between 2002 and 2021, the number of incident lung cancer cases each year increased from 3708 to 5,211, with a total of 87,259 incident cases in all years combined (Table 2). More than 60% of the incident cases were male, and close to 80% of the incident cases were aged 60 or above (Table 2). Similar to the age, and sex distribution of prevalent cases (Table 1), the percentage of male incident cases gradually decreased over the years, from 67.6% in 2002 to 59.8% in 2021, and the percentage of different age groups remained relatively stable (Table 2). The crude incidence (per 100,000 persons) increased from 55.0 to 70.3, with an APC of 1.23% (95% CI: 1.02 to 1.44; p < 0.001) (Fig. 2A) (Supplementary Table S4). Age-standardised incidence (per 100,000 persons) on the other hand decreased from 42.9 to 33.2, with an APC of −1.32% (95% CI: −1.49 to −1.15; p < 0.001) (Fig. 2A) (Supplementary Table S4).

**Table 2:** Age and sex distribution of incident lung cancer cases between 2002 and 2021.

Year	Total number of incident cases	By sex		By age group			
		Male (%)	Female (%)	Age < 40 (%)	Age 40–59 (%)	Age 60–79 (%)	Age > 79 (%)
2002	3708	2506 (67.6)	1202 (32.4)	52 (1.4)	663 (17.9)	2266 (61.1)	727 (19.6)
2003	3549	2461 (69.3)	1088 (30.7)	42 (1.2)	733 (20.7)	2110 (59.5)	664 (18.7)
2004	3806	2538 (66.7)	1268 (33.3)	50 (1.3)	737 (19.4)	2239 (58.8)	780 (20.5)
2005	3884	2583 (66.5)	1301 (33.5)	51 (1.3)	771 (19.9)	2261 (58.2)	801 (20.6)
2006	3911	2698 (69)	1213 (31)	44 (1.1)	846 (21.6)	2215 (56.6)	806 (20.6)
2007	3917	2600 (66.4)	1317 (33.6)	57 (1.5)	794 (20.3)	2185 (55.8)	881 (22.5)
2008	3961	2602 (65.7)	1359 (34.3)	38 (1)	827 (20.9)	2198 (55.5)	898 (22.7)
2009	4298	2821 (65.6)	1477 (34.4)	49 (1.1)	925 (21.5)	2278 (53)	1046 (24.3)
2010	4330	2852 (65.9)	1478 (34.1)	38 (0.9)	930 (21.5)	2299 (53.1)	1063 (24.5)
2011	4293	2812 (65.5)	1481 (34.5)	40 (0.9)	965 (22.5)	2256 (52.6)	1032 (24)
2012	4542	2941 (64.8)	1601 (35.2)	35 (0.8)	962 (21.2)	2409 (53)	1136 (25)
2013	4428	2866 (64.7)	1562 (35.3)	47 (1.1)	921 (20.8)	2347 (53)	1113 (25.1)
2014	4478	2870 (64.1)	1608 (35.9)	40 (0.9)	955 (21.3)	2376 (53.1)	1107 (24.7)
2015	4563	2823 (61.9)	1740 (38.1)	45 (1)	921 (20.2)	2470 (54.1)	1127 (24.7)
2016	4681	2977 (63.6)	1704 (36.4)	36 (0.8)	989 (21.1)	2521 (53.9)	1135 (24.2)
2017	4797	3005 (62.6)	1792 (37.4)	45 (0.9)	938 (19.6)	2639 (55)	1175 (24.5)
2018	4874	3049 (62.6)	1825 (37.4)	46 (0.9)	938 (19.2)	2734 (56.1)	1156 (23.7)
2019	5171	3240 (62.7)	1931 (37.3)	40 (0.8)	961 (18.6)	2943 (56.9)	1227 (23.7)
2020	4857	2985 (61.5)	1872 (38.5)	47 (1)	816 (16.8)	2895 (59.6)	1099 (22.6)
2021	5211	3117 (59.8)	2094 (40.2)	33 (0.6)	915 (17.6)	3042 (58.4)	1221 (23.4)

In males, the age-standardised incidence (per 100,000 persons) showed a decreasing trend with an APC of −1.92% (95% CI: −2.12 to −1.73; p < 0.001), while in females, the incidence remained stable over the years (p for APC = 0.306) (Fig. 2B) (Supplementary Table S5). Although with a decreasing trend, the incidence in males was consistently higher, ranging from 55.4 to 38.4, compared to females which stayed around 26 (Fig. 2B) (Supplementary Table S4).

When stratified by age groups, for populations aged between 60 and 79, and above 79, the incidence (per 100,000 persons) showed a decreasing trend with an AAPC of −1.98% (95% CI: −2.38 to −1.59; p < 0.001) and −1.94% (95% CI: −2.72 to −1.15; p < 0.001), respectively (Fig. 2C) (Supplementary Table S5). For populations aged between 40 and 59, and aged below 40, the overall incidence remained relatively constant (p for AAPC/APC = 0.057 and 0.642, respectively) (Fig. 2C) (Supplementary Table S5). Since 2010, there was an apparent drop in incidence in the above 79 age group which the APC decreased from −0.67% (95% CI: −2.31 to 0.99; p = 0.401) to −2.85% (95% CI: −3.70 to −1.99; p < 0.001) (Fig. 2C) (Supplementary Table S5). The decreasing trend in the 60–79 age group on the other hand slowed down since 2015, with the APC increasing from −2.36% (95% CI: −2.73 to −1.99; p < 0.001) to −1.16% (95% CI: −2.25 to −0.05; p = 0.042) (Fig. 2C) (Supplementary Table S5). On the other hand, for ages 40–59, there was a slight increase between 2002 and 2011 (APC: 2.16; 95% CI: 0.86 to 3.47; p = 0.003), which then flattened out (Fig. 2C) (Supplementary Table S5). Over the years, lung cancer incidence was consistently higher in the older age groups, with incidence ranged between 283.1 and 456.4 for those aged above 79, and between 181.1 and 263.7 for those aged between 60 and 79 (Fig. 2C) (Supplementary Table S4). The incidence in younger age groups remained low with only an average of 37.6 for aged between 40 and 59, and an average of 1.3 for aged below 40 (Fig. 2C) (Supplementary Table S4).

### Trends in 1-year and 5-year relative period survival by sex, age, and comorbidity severity

**Fig. 3:**
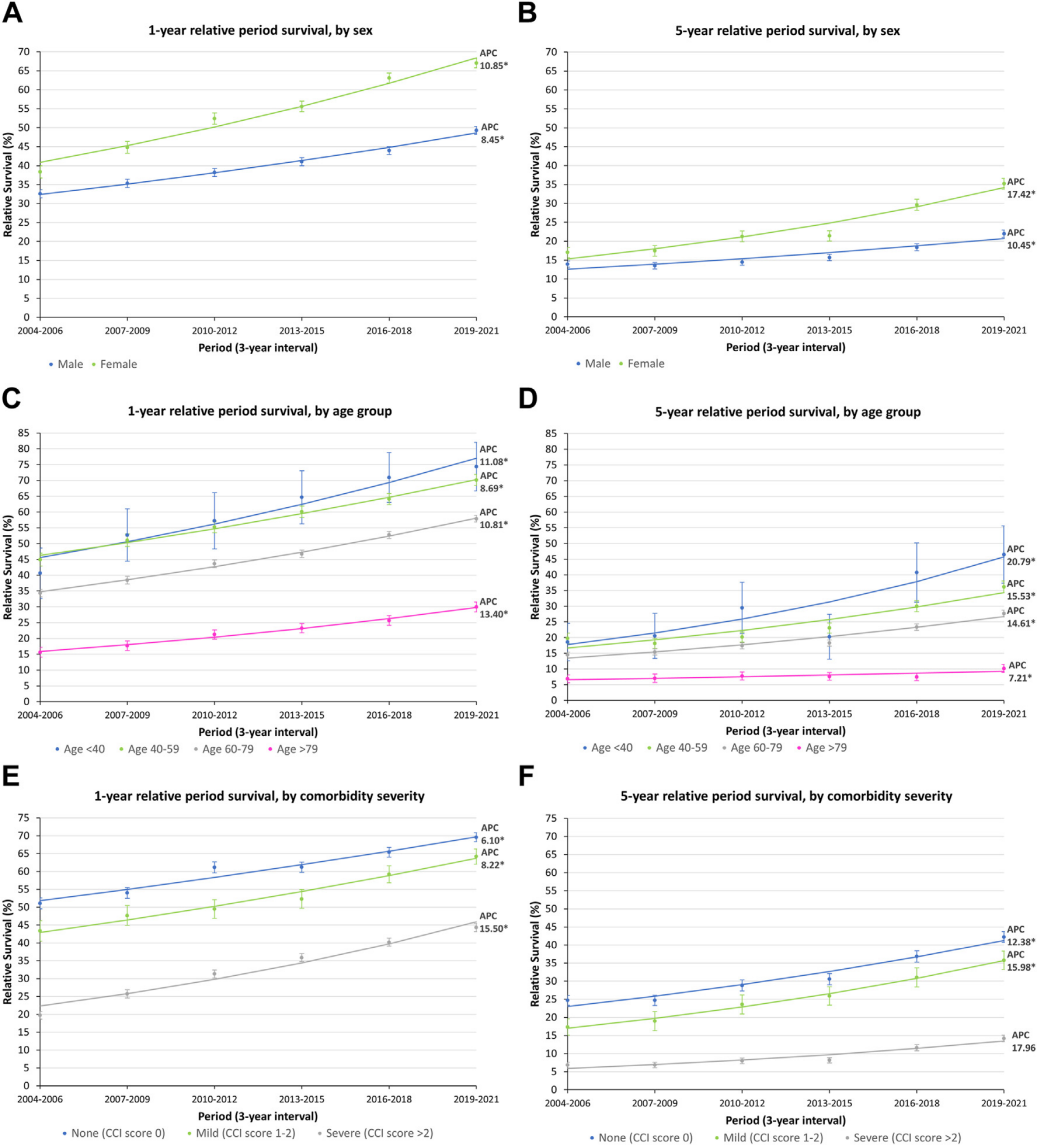
**The trends in lung cancer 1-year/5-year relative period survival between 2004 and 2021.** (A) 1-year relative period survival by sex, (B) 5-year relative period survival by sex; (C) 1-year relative period survival by age group, (D) 5-year relative period survival by age group; (E) 1-year relative period survival by comorbidity severity, (F) 5-year relative period survival by comorbidity severity. AAPC: average annual percent change, APC: annual percent change, CCI: Charlson Comorbidity Index. ∗Indicates significant (p ≥ 0.05) APC/AAPC.

The 1-year and 5-year relative period survivals for different sex, age groups and comorbidity levels all showed an increasing trend between 2004 and 2021, with a drastic decrease in survival between 1-year and 5-year (Fig. 3). Females consistently showed a better survival across the years, with 5-year survival increased from 17.0% to 35.2% (APC: 17.42%; 95% CI: 10.99 to 24.22; p = 0.001), compared to males, with 5-year survival increased from 14.0% to 22.0% (APC: 10.45%; 95% CI: 5.06 to 16.11; p = 0.005) (Fig. 3A and B) (Supplementary Table S6). Among different age groups, older age groups showed worse survival (Fig. 3C and D). The lowest survival was observed in those aged above 79, with less than 30% 1-year survival and 10% 5-year survival, compared to other age groups with 1-year survival ranging from 34.3% to 74.3%, and 5-year survival ranging from 14.5% to 46.5% (Supplementary Table S6). Not only patients aged above 79 had the lowest survival, but they also showed the least improvement in 5-year survival over the years, with an APC of 7.21% (95% CI: 0.23 to 14.68; p = 0.045), which was less than half of the annual percentage increases observed in other age groups (Fig. 3D) (Supplementary Table S7). For different comorbidity severity, although all showed improvement over the years (Fig. 3E and F), the survival of patients with severe comorbidity remained the lowest, with 5-year survival ranged from 6.8% to 14.1%, compared to 17.3%–42.2% in those with mild or no comorbidity (Fig. 3F) (Supplementary Table S6). The baseline comorbidity severity of the survival cohort was shown in Supplementary Table S8.

### Comparison with incidence trends reported by the HKCR

**Fig. 4:**
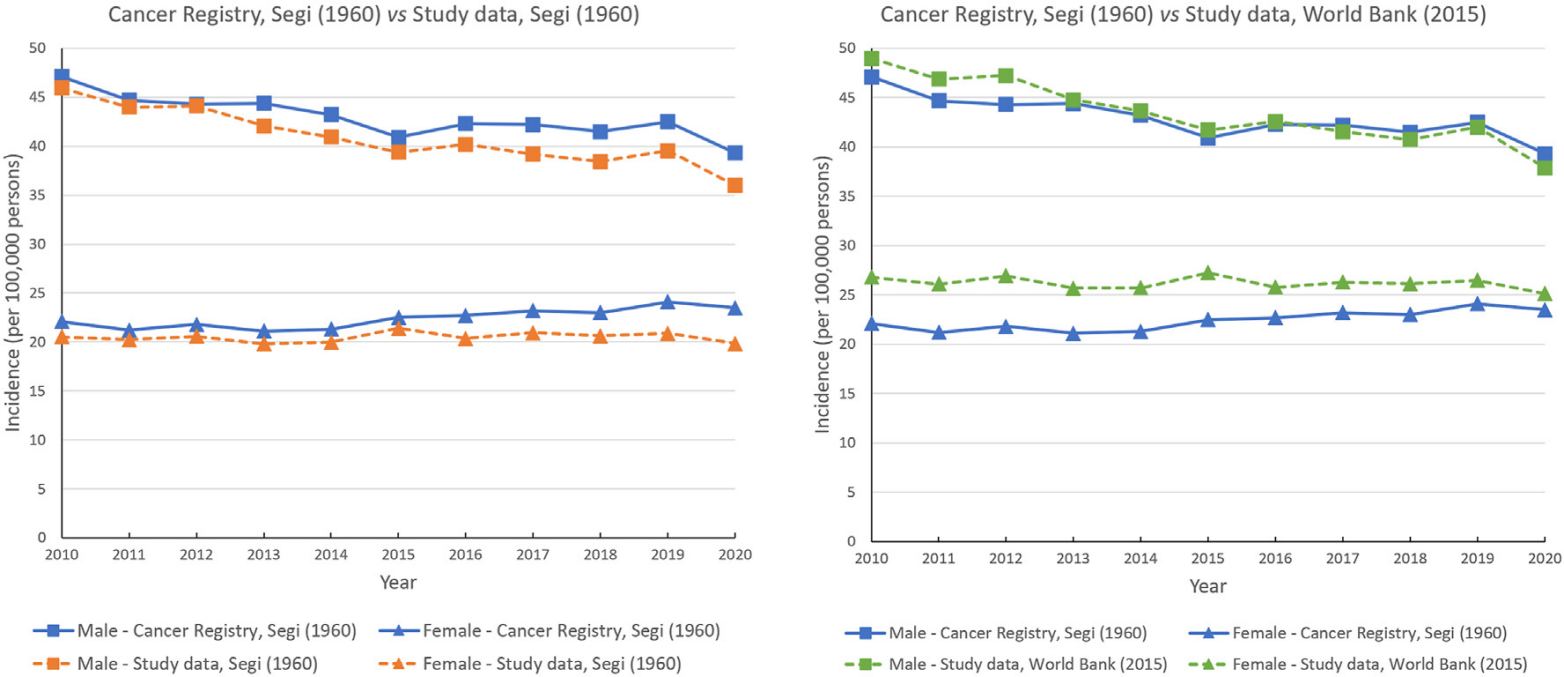
**Comparison with the sex-specific age-standardised incidence reported by the Hong Kong Cancer Registry**.

Sex-specific lung cancer incidence was age-standardised to the Segi (1960)^21^ and the World Bank (2015)^9^ standard populations, separately. For both standards, the incidence trends were highly comparable to the trends reported by the HKCR which used the Segi (1960) population (Fig. 4). Compared to the annual age-standardised incidence reported by the HKCR, the age-standardised incidence reported in the present study was generally lower when using the 1960 population, and higher when using the 2015 population (Fig. 4).

## Discussion

To our knowledge, this population-based study provided the most up-to-date and comprehensive evaluation of the changing epidemiology of lung cancer in the past two decades in a Chinese population. Since 2008, the overall 10-year limited-duration prevalence of lung cancer has been increasing in Hong Kong. Females and males showed similar increasing trends, while the trends among different age groups were mixed. For lung cancer incidence, the crude incidence has been increasing since 2002, and an opposite trend was observed after age standardisation. Males consistently showed a higher age-standardised incidence with a decreasing trend over the years, while the incidence in females was lower and remained relatively stable. Compared to younger age groups, older age groups showed a more apparent decreasing trend. For relative period survival of lung cancer patients, both 1-year and 5-year survivals have shown continuous improvement since 2004, although remained low. Among different sub-groups, being females, young, and having low comorbidity have consistently shown better survival.

Over the past two decades, the disease burden of lung cancer in Hong Kong has been increasing. The overall prevalence and the crude incidence have increased by 43% and 25%, respectively. Opposite decreasing incidence trends were observed after age-standardisation. The age-specific prevalence also showed similar attenuation in increase. Given the higher incidence observed in the older population, the increase in crude incidence could be attributed to an ageing population. In fact, between 2002 and 2021, the proportion of people aged 60 or above in Hong Kong has increased from 15.1% to 27.9%.^8^ Decreasing trends in age-standardised lung cancer incidence were also observed in China and the US, until recent years when the trends went up due to the implementation of lung cancer screening programmes.^22^ The trend in age-standardised incidence in the UK on the other hand remained relatively stable over the past decade.^23^

The trends in age-standardised incidence showed a disparity between females and males. The incidence in males showed a decreasing trend over the years, while the incidence in females remained stable. Such disparity could be reflective of the change in smoking habits. Cigarette smoking has been a well-established risk factor for lung cancer with an attributed risk of 45.8% in males and 6.2% in females in Hong Kong.^24^ Over the past decades, the legislation for tobacco control^25^ and the establishment of the Tobacco Control Office^26^ in the 1980s to early 2000s, together with the increased tobacco tax and the enforcement of no-smoking areas in more recent years,^25^^,^^27^ have successfully reduced the number of prevalent smokers in Hong Kong. The prevalence of daily cigarette (excluding e-cigarette) smokers aged 15 and over has been reducing steadily from 23.3% in 1982 to 10.2% in 2019,^28^ the decrease of which concurs with the decrease in age-standardised incidence of lung cancer. Among females and males, although the prevalence of male smokers was consistently higher than female smokers, the reduction over time was more prominent—decreasing from 39.7% in 1982 to 18.1% in 2019 for males, compared to the low and stable prevalence of around 4% for females.^28^ This difference in trends mirrored the greater decline in lung cancer incidence in males and the stable incidence in females (Supplementary Fig. S2). In fact, it was shown that smoking and second-hand smoke exposure are not the main causes of lung cancer for women in Hong Kong, and hence the relatively low attributed risk of smoking observed in females.^29^ A similar correlation between sex-specific smoking prevalence trends^30^, ^31^, ^32^ and sex-specific lung cancer incidence trends was also observed in China, the US, and the UK.^22^^,^^33^ Of note, these countries all showed a decreasing or overall stable trend in male and female incidences, except for the UK females who showed an increasing trend,^33^ which corresponded to the increase in female smokers between the 1950s and 1970s in the UK.^32^ Air pollutants, although being another risk factor for incident lung cancer,^34^ are unlikely to explain the incidence disparity observed between females and males. The association between fine particles (PM2.5) and lung cancer risk was shown to be stronger in females with a higher attributable fraction than in males.^35^ Same for the cooking emissions. Studies have shown that poor kitchen ventilation and exposure to cooking fumes were associated with higher lung cancer risks among Chinese women^36^^,^^37^ and women generally spend more time in the kitchen than men. If air pollution and cooking emissions were the key factors behind the sex disparity, the female incidence would have been higher than the male incidence. Since male incidence was consistently higher, this suggested that the key factor behind the observed sex disparity was probably the difference in smoking habits.

For different age groups, overall decreasing trends in incidence were observed in age groups above 60 (which constituted around 80% of all the lung cancer cases). For those aged above 79, a greater decline was observed in recent years. This shift could be attributed to the progressive tobacco controls implemented through legislation and the establishment of the Tobacco Control Office in the 1990s and early 2000s.^25^^,^^26^ However, for those aged between 60 and 79, although still showed an overall decreasing trend, the decline in incidence slightly slowed down in recent years. The increasing use of e-cigarettes and heated tobacco products would not be an explanation since their uses in Hong Kong have not gained popularity until recent years and the prevalence remained low, with only 0.1% and 0.2% of the population in 2019 being daily smokers, respectively.^38^ Given more than 50% of the incident cases of lung cancer belong to this 60-79 age group, close monitoring of the trend in future years is warranted. Moreover, although the 40–59 age group showed an overall stable trend over the years, there was a slight increase in incidence between 2002 and 2011. This increase could be reflective of the transient increase in daily cigarette smokers from the early 1990s to the early 2000s among the 20–29 and 30–39 age groups.^28^

Advancements in cancer imaging techniques and treatment strategies in the past decades have contributed to the continuous improvement of lung cancer survival. The introduction of positron emission tomography (PET) and positron emission tomography–computed tomography (PET-CT) allowed more accurate imaging and staging of the disease and helped physicians in tailoring the optimal treatment strategies. The increasing use of precise radiotherapy deliveries, such as (IMRT) and stereotactic ablative radiotherapy (SABR), also increased the survival of lung cancer patients. The most notable advancements in recent years are the developments of targeted therapy and immunotherapy for non-small cell lung cancer (NSCLC). The landmark discovery of targetable oncogene–epidermal growth factor receptor (EGFR), and the use of EGFR-tyrosine kinase inhibitors have significantly improved the survival of advanced-stage lung NSCLC patients harbouring the corresponding mutations.^39^ More recently, the discovery of programmed cell death protein 1/programmed death ligand 1 (PD-1/PD-L1) inhibitors further provided an effective treatment option for NSCLC patients expressing high levels of PD-L1.^40^ The improved survival brought about by the introduction of genetic testing in 2009 and the approval of immunotherapy for NSCLC in 2015 in Hong Kong could have contributed to the upward shift in lung cancer prevalence since 2016–2017.

Although there was consistent improvement in lung cancer survival over the years, disparity existed among different sex, age, and comorbidity sub-groups. Worse survival was observed in older patients and patients with more comorbid conditions, which could readily be attributed to the high mortality rate among these patients. Particularly, the improvement in survival among patients aged above 79 was almost negligible. This could be reflective of the naturally high mortality rate and the preference for palliative treatments in this age group. Cost-effectiveness studies would provide further insight into the resource allocation among patients of the oldest old. Moreover, females consistently showed longer survival than males. The same disparity was also observed in China,^41^ the UK,^42^ and the US.^43^ Never smokers were shown to have better lung cancer survival than ex-smokers and current smokers.^44^ The better survival in females could be reflective of the lower prevalence of female smokers. Further study is needed to examine how lifestyle and genetic differences could explain the disparity.

The results from the present study highlighted the potential need for adjustments in clinical management and related policies. Although age-standardised lung cancer incidence showed a decreasing trend over the years, the prevalence of lung cancer patients and survivors continued to rise due to the ageing population and improved survival. Cancer is considered a chronic disease. Cancer survivors have been shown to associate with higher comorbidity^45^ and the prevalence of comorbidity in lung cancer patients was amongst the highest compared to other cancers.^46^ Comorbidity in cancer survivors results in higher annual medical expenditures and annual lost productivity.^45^ Adaptation and adjustment to disease management and resource allocation would be warranted to tackle the increasing burden of disease on society. On the other hand, from the patient’s perspective, despite continuous improvement in lung cancer survival, both short- and long-term survival remained extremely low. Large randomised clinical trials from the US (the National Lung Screening Trial [NLST])^47^ and Europe (the Nederlands–Leuvens Longkanker Screenings Onderzoek [NELSON])^48^ have shown that screening by low-dose computed tomography (LDCT) detects lung cancer at an earlier stage and reduces lung cancer death. Public lung cancer screening programmes have been implemented in many countries, including China,^49^ the UK,^50^ and the US.^51^ However, in many countries and regions, like Hong Kong, screening programme is still currently not available. Therefore, the implementation of lung cancer screening programme could potentially improve further the survival of lung cancer patients.

The present study is highly generalizable and representative of the lung cancer population in Hong Kong. The CDARS is a routinely updated healthcare database of the public healthcare system in Hong Kong. Unlike many claim databases, the CDARS is non-selective in terms of patients’ age, sex, and socioeconomic status, and it covers approximately 90% of lung cancer patients in Hong Kong.^4^ Death records are highly complete due to the linkage to the government death registry in Hong Kong. Therefore, loss to follow-up with unknown death status is unlikely, even when patients move out into the private sector. Compared to the HKCR which reports only summary statistics on incidence and mortality, the longitudinal nature of the CDARS data allows the estimation of additional epidemiological measures, such as prevalence and survival. Therefore, the CDARS would be a better data source for studying lung cancer epidemiology. Moreover, the 1960 standard population used by the HKCR is a very “young” population. The generally lower age-standardised incidence yielded from the 1960 standard population compared to the 2015 one suggested that the estimates reported by the HKCR should be interpreted with caution.

There are limitations to the present study. First, the study does not distinguish between histological groups, cancer stages and genetic mutation status. As a reference, in Hong Kong in 2019, the majority (82.3%) of the incident lung cancer cases were NSCLC, and adenocarcinoma comprised 61.2% of the NSCLC cases.^2^ A 57.3% of the lung cancer cases were stage IV.^2^ A 44.5% of the tested patients were positive for EGFR mutation.^2^ Treatment options, management guidelines and prognosis vary between different types and stages of lung cancer. It would be clinically important to look additionally at the trends by different sub-groups of lung cancer in future studies. Second, since we could not ascertain the date the cancer was “cured”, which is an inherent limitation in most cancer studies, the present study adopted the limited-duration method to estimate prevalence. The limited-duration prevalence was calculated as the proportion of people alive in a certain year who had lung cancer diagnosed within a pre-defined time period. Therefore, the use of a 10-year period would include both ongoing cancer patients, those in remission, and cancer-free survivors. Cancer is generally considered a chronic disease and cancer survivors are associated with higher comorbidity.^45^ The inclusion of cancer survivors would be informative about the resources burden on the healthcare system. Sensitivity analysis was also conducted using shorter periods (5- and 3-year) to limit the prevalent cases to those with current cancer and showed comparable trends in prevalence. Nonetheless, the interpretation of the limited-duration prevalence should be cautious as it included not only ongoing cancer patients. On the other hand, a longer period was not used because the CDARS database is a relatively young database and medical records from early years are not complete. Therefore, there was a risk of excluding prevalent cases diagnosed before the 10-year period. However, since the long-term survival of lung cancer is low, the majority of the prevalent cases should have been captured within the 10-year period and it was unlikely to have a significant impact on the estimated trends. Third, lung cancer patients diagnosed and followed in the private sector would be missing in the CDARS. However, they constituted only approximately 10% of all lung cancer patients in Hong Kong.^4^ Based on the highly comparable incidence estimates between the present study and the HKCR, the incidence and prevalence estimates in the present study were highly representative of the lung cancer population in Hong Kong. In terms of survival, public healthcare in Hong Kong covers a comprehensive list of cancer therapies for patients who cannot afford private healthcare. The doctors in the public sector and the private sector are equally qualified. The standard of care in public and private healthcare is comparable and significant difference in survival is not expected. Therefore, the approximately 10% missing patients from the CDARS would not have a significant impact on the study results. Fourth, sub-group analysis by different levels of comorbidity was only performed for survival trends because there was a lack of comorbidity data from the mid-year population for incidence and prevalence analyses. Last, the present study did not evaluate other indices for disease burden, such as the disability-adjusted life year (DALY) and the economic burden. Future studies assessing additional indices would provide a more comprehensive view of the disease burden.

To conclude, over the past two decades, the prevalence and crude incidence of lung cancer have been increasing in Hong Kong. When standardised by age, the incidence showed a decreasing trend, suggesting that an ageing population was a key factor in the increasing disease burden. Although both short- and long-term survivals showed improvement over the years, they remained low, highlighting the potential need for interventions. Disparity in incidence and survival trends were observed among different sex and age groups. Further study explaining the difference in sex-specific trends, as well as on different histological groups and stages of lung cancer is warranted.

## Contributors

PCA and CLC contributed to the literature search, study design, and writing of the manuscript. PCA contributed to the data analysis and data interpretation of the manuscript. PCA and CLC had directly accessed and verified the underlying data reported in the manuscript. AWL, VHL, ICW, RYH and CLC contributed to the discussion and reviewed/edited the manuscript. All contributing authors were from the academic team. All authors had full access to all the data in the study and accept responsibility to submit for publication.

## Data sharing statement

All study data analysed during this study were used under license by the Hospital Authority (HA) of Hong Kong. Restrictions apply. The study data are not publicly available.

## Editor note

The Lancet Group takes a neutral position with respect to territorial claims in published maps and institutional affiliations.

## Declaration of interests

PCA, AWL, VHL, ICW, and CLC declare that they have no competing interests. RYH declares that in the past 36 months, all outside of the submitted work, the author received grants from AstraZeneca, Bristol Myers Squibb, Corvus, Eisai, Eli Lilly, Janssen, Merck Sharp & Dohme, Novartis, and Roche; consulting fees from Amgen, AstraZeneca, Bristol Myers Squibb, Eisai, Eli Lilly, Janssen, Merck Sereno, Merck Sharp & Dohme, Novartis, Pfizer, Roche, Takeda, and Zai Lab; honoraria from AstraZeneca, Eli Lilly, Janssen, Merck Sharp & Dohme, and Novartis. The study sponsor, ITC, did not interfere with authors’ access to study’s data, or that interfere with their ability to analyse and interpret the data and to prepare and publish manuscripts independently.
