# Supplementary material for: The trends in lung cancer prevalence, incidence, and survival in Hong Kong over the past two decades (2002–2021): a population-based study
Source: Lancet Reg Health West Pac. 2024 Feb 16;45:101030. doi: 10.1016/j.lanwpc.2024.101030 (PMC10882113; doi:10.1016/j.lanwpc.2024.101030)
Supplement: Supplementary Figs. S1 and S2 and Tables S1–S8 [file mmc1.docx]

Content:

Supp Figure 1. Sensitivity analysis using 5-year and 3-year limited-duration prevalence.

Supp Figure 2. Comparison between the trend in smoking prevalence and the trend in age-standardised lung cancer incidence.

Supp Table 1. ICD-9-CM definitions of comorbidity for the Charlson Comorbidity Index (CCI) score

Supp Table 2. 10-year limited-duration prevalence (per 100,000 persons) of lung cancer by year.

Supp Table 3. Joinpoint regression analysis of the trend in lung cancer prevalence between 2008 and 2021.

Supp Table 4. Incidence (per 100,000 persons) of lung cancer by year.

Supp Table 5. Joinpoint regression analysis of the trend in lung cancer incidence between 2002 and 2021.

Supp Table 6. Relative period survival (%) of lung cancer by year period.

Supp Table 7. Annual Percent Change of lung cancer survival between 2004 and 2021.

Supp Table 8. Baseline comorbidity severity of the survival cohort.

**Supp Figure 1. Sensitivity analysis using 5-year and 3-year limited-duration prevalence.**

**
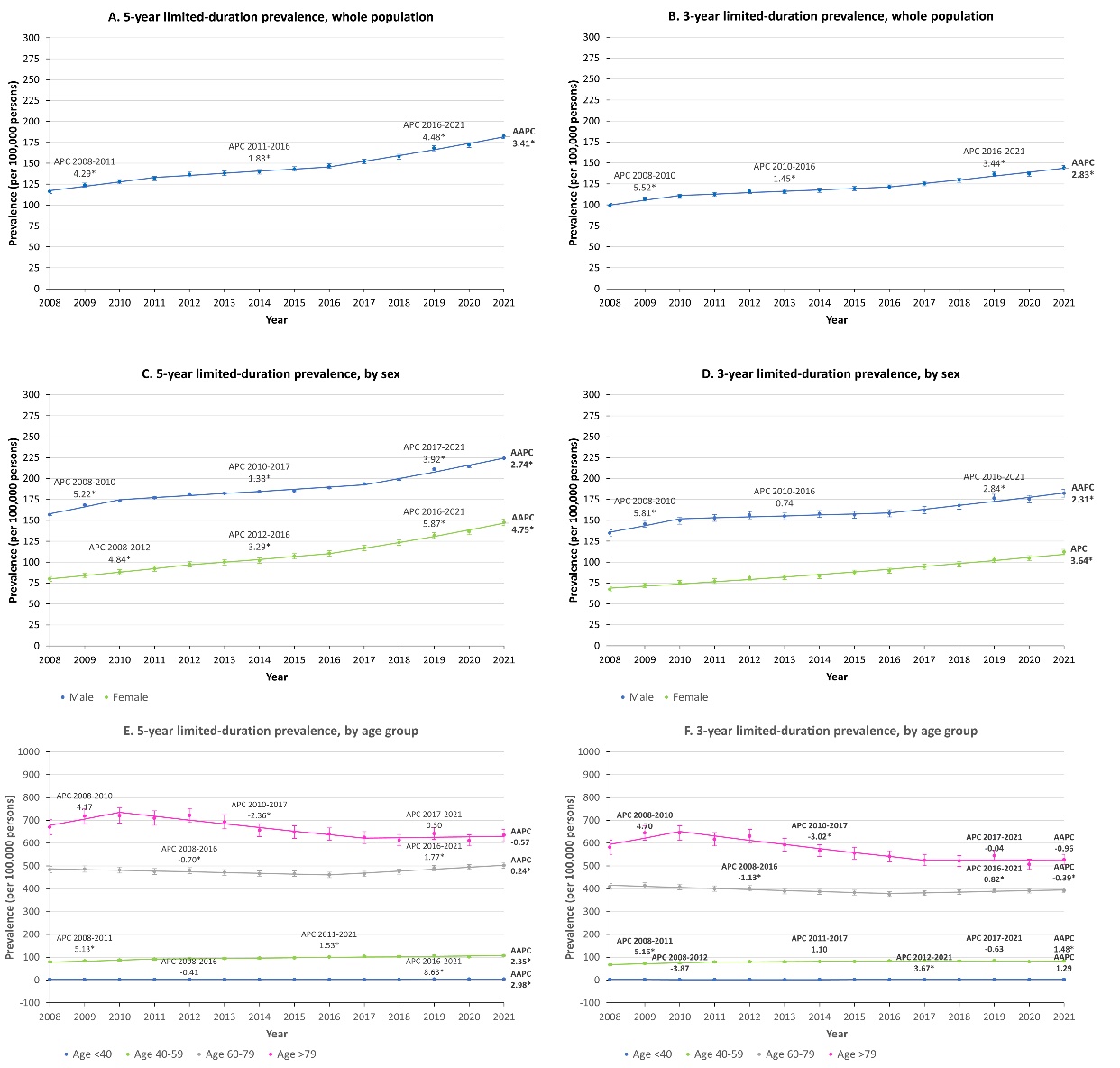
**

AAPC: average annual percent change, APC: annual percent change.

* Indicates significant (p≥0.05) APC/AAPC.

**Supp Figure 2. Comparison between the trend in smoking prevalence and the trend in age-standardised lung cancer incidence.**


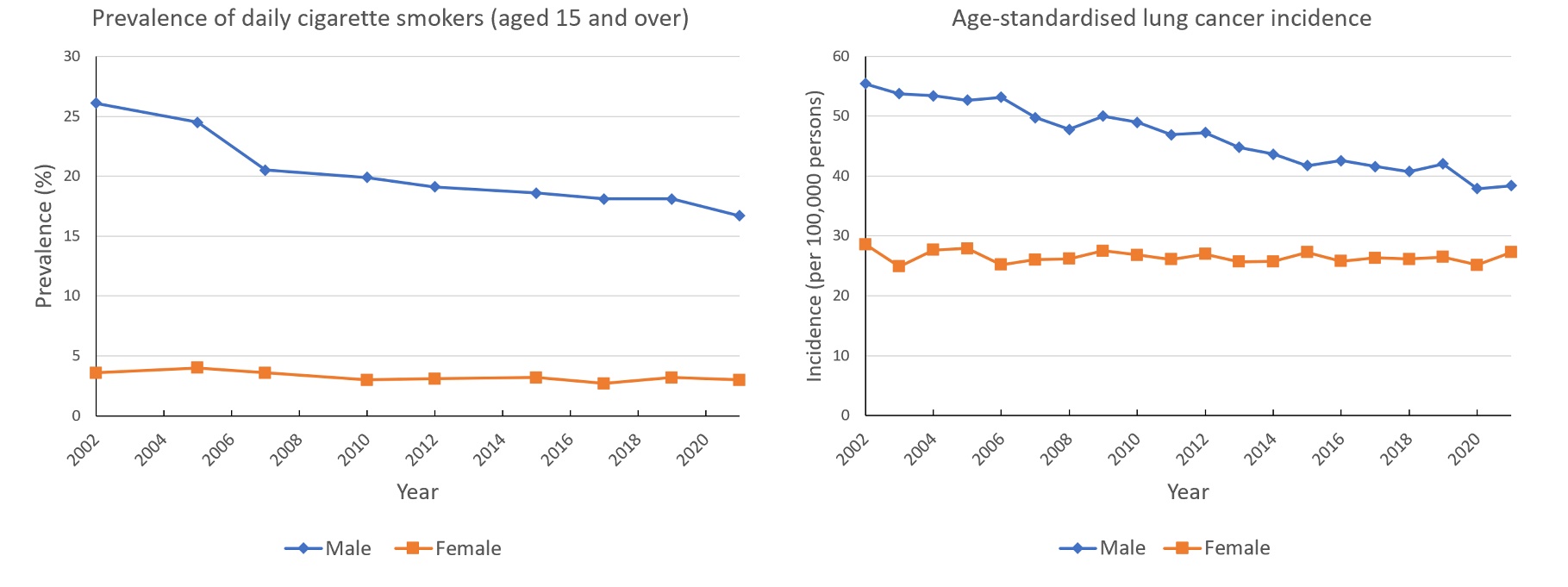


**Supp Table 1. ICD-9-CM definitions of comorbidity for the Charlson Comorbidity Index (CCI) score**

| **Comorbidities** | **ICD-9-CM definitions** |
| --- | --- |
| Myocardial infarction | 410.x, 412.x |
| Congestive heart failure | 398.91, 402.01, 402.11, 402.91, 404.01, 404.03, 404.11, 404.13, 404.91, 404.93, 425.4-425.9, 428.x |
| Peripheral vascular disease | 093.0, 437.3, 440.x, 441.x, 443.1-443.9, 47.1, 557.1, 557.9, V43.4 |
| Cerebrovascular disease | 362.34, 430.x-438.x |
| Dementia | 290.x, 294.1, 331.2 |
| Chronic pulmonary disease | 416.8, 416.9, 490.x-505.x, 506.4, 508.1, 508.8 |
| Rheumatic disease | 446.5, 710.0-710.4, 714.0 714.2, 714.8, 725.x |
| Peptic ulcer dise | 531.x-534.x |
| Mild liver disease | 070.22, 070.23, 070.32, 070.33, 070.44, 070.54, 070.6, 070.9, 570.x, 571.x, 573.3, 573.4, 573.8, 573.9, V42.7 |
| Diabetes without chronic complication | 250.0-250.3, 250.8, 250.9 |
| Diabetes with chronic complication | 250.4-250.7 |
| Hemiplegia or paraplegia | 334.1, 342.x, 343.x, 344.0 344.6, 344.9 |
| Renal disease | 403.01, 403.11, 403.91, 404.02, 404.03, 404.12, 404.13, 404.92, 404.93, 582.x, 583.0-583.7, 585.x, 586.x, 588.0, V42.0, V45.1, V56.x |
| Any malignancy, including lymphoma and leukemia, except malignant neoplasm of skin, and malignant neoplasm of lung | 140.x-161.x, 163.x-172.x, 174.x-195.8, 200.x-208.x, 238.6 |
| Moderate or severe liver disease | 456.0-456.2, 572.2-572.8 |
| Metastatic solid tumour | 196.x-199.x |
| AIDS/HIV | 042.x-044.x |

**Supp Table 2. 10-year limited-duration prevalence (per 100,000 persons) of lung cancer by year**

| Year |  | Whole population |  | By sex | |  | By age group | | | |
| --- | --- | --- | --- | --- | --- | --- | --- | --- | --- | --- |
|  |  |  |  | Male | Female |  | Age <40 | Age 40-59 | Age 60-79 | Age >79 |
| 2008 |  | 153.4 |  | 208.6 | 103.8 |  | 3.7 | 98.5 | 639.6 | 928.1 |
| 2009 |  | 157.3 |  | 214.2 | 106.6 |  | 3.5 | 101.4 | 620.0 | 961.3 |
| 2010 |  | 158.6 |  | 214.8 | 109.0 |  | 3.2 | 102.9 | 598.6 | 936.8 |
| 2011 |  | 159.9 |  | 215.3 | 111.3 |  | 3.3 | 105.8 | 578.4 | 898.7 |
| 2012 |  | 164.7 |  | 219.7 | 116.9 |  | 3.0 | 108.0 | 581.5 | 898.1 |
| 2013 |  | 167.1 |  | 220.2 | 121.1 |  | 3.3 | 108.7 | 571.4 | 873.3 |
| 2014 |  | 170.5 |  | 223.9 | 124.5 |  | 3.5 | 111.2 | 569.5 | 837.1 |
| 2015 |  | 175.3 |  | 226.2 | 131.6 |  | 3.2 | 112.9 | 572.0 | 834.6 |
| 2016 |  | 179.8 |  | 231.4 | 135.7 |  | 3.0 | 117.5 | 566.1 | 824.9 |
| 2017 |  | 186.9 |  | 237.3 | 144.2 |  | 3.4 | 120.3 | 573.0 | 810.4 |
| 2018 |  | 195.1 |  | 245.6 | 152.6 |  | 3.7 | 120.3 | 588.7 | 797.1 |
| 2019 |  | 206.1 |  | 257.3 | 163.2 |  | 4.0 | 124.4 | 603.6 | 817.7 |
| 2020 |  | 213.6 |  | 262.5 | 172.5 |  | 4.6 | 122.8 | 617.5 | 781.0 |
| 2021 |  | 228.7 |  | 276.3 | 188.7 |  | 4.5 | 128.2 | 632.5 | 813.5 |

**Supp Table 3. Joinpoint regression analysis of the trend in lung cancer prevalence between 2008 and 2021**

| **A) Whole population** | | | | | |
| --- | --- | --- | --- | --- | --- |
|  | Measure | Year segment | Estimate (%) | 95% CI | p |
|  | AAPC | 2008-2021 | 3.08 | 2.85, 3.31 | <0.001 |
|  | APC | 2008-2016 | 1.91 | 1.63, 2.20 | <0.001 |
|  |  | 2016-2021 | 4.97 | 4.45, 5.49 | <0.001 |
|  |  |  |  |  |  |
| **B) By sex** |  |  |  |  |  |
|  | Measure | Year segment | Estimate (%) | 95% CI | p |
| Male | AAPC | 2008-2021 | 2.11 | 1.90, 2.31 | <0.001 |
|  | APC | 2008-2016 | 1.13 | 0.88, 1.37 | <0.001 |
|  |  | 2016-2021 | 3.70 | 3.23, 4.16 | <0.001 |
| Female | AAPC | 2008-2021 | 4.62 | 4.13, 5.11 | <0.001 |
|  | APC | 2008-2011 | 2.33 | 0.53, 4.16 | 0.019 |
|  |  | 2011-2017 | 4.19 | 3.45, 4.93 | <0.001 |
|  |  | 2017-2021 | 7.03 | 6.13, 7.95 | <0.001 |
|  |  |  |  |  |  |
| **C) By age group** | | | | | |
|  | Measure | Year segment | Estimate (%) | 95% CI | P |
| Age <40 | AAPC | 2008-2021 | 2.37 | 0.66, 4.10 | 0.006 |
|  | APC | 2008-2016 | -1.45 | -3.38, 0.52 | 0.130 |
|  |  | 2016-2021 | 8.79 | 4.60, 13.14 | 0.001 |
| Age 40-59 | AAPC | - | - | - | - |
|  | APC | 2008-2021 | 1.98 | 1.82, 2.13 | <0.001 |
| Age 60-79 | AAPC | 2008-2021 | -0.10 | -0.34, 0.14 | 0.413 |
|  | APC | 2008-2011 | -3.19 | -4.01, -2.36 | <0.001 |
|  |  | 2011-2016 | -0.56 | -1.07, -0.05 | 0.035 |
|  |  | 2016-2021 | 2.27 | 1.95, 2.59 | <0.001 |
| Age >79 | AAPC | 2008-2021 | -1.36 | -1.97, -0.75 | <0.001 |
|  | APC | 2008-2017 | -1.91 | -2.51, -1.32 | <0.001 |
|  |  | 2017-2021 | -0.10 | -1.96, 1.80 | 0.909 |

AAPC: average annual percent change, APC: annual percent change, CI: confidence interval.

**Supp Table 4. Incidence (per 100,000 persons) of lung cancer by year**

| Year |  | Whole population | |  | By sex* | |  | By age group | | | |
| --- | --- | --- | --- | --- | --- | --- | --- | --- | --- | --- | --- |
|  |  | Crude | Age-standardised* |  | Male | Female |  | Age <40 | Age 40-59 | Age 60-79 | Age >79 |
| 2002 |  | 55.0 | 42.9 |  | 55.4 | 28.6 |  | 1.4 | 33.2 | 263.7 | 456.4 |
| 2003 |  | 52.7 | 40.3 |  | 53.7 | 24.9 |  | 1.2 | 35.2 | 245.9 | 392.9 |
| 2004 |  | 56.1 | 41.4 |  | 53.4 | 27.7 |  | 1.4 | 34.1 | 257.4 | 433.8 |
| 2005 |  | 57.0 | 41.0 |  | 52.6 | 27.9 |  | 1.5 | 34.5 | 257.1 | 420.7 |
| 2006 |  | 57.0 | 40.0 |  | 53.2 | 25.2 |  | 1.3 | 37.1 | 248.2 | 396.3 |
| 2007 |  | 56.6 | 38.6 |  | 49.7 | 26.0 |  | 1.6 | 34.4 | 235.5 | 406.4 |
| 2008 |  | 56.9 | 37.7 |  | 47.8 | 26.2 |  | 1.1 | 35.4 | 229.4 | 393.7 |
| 2009 |  | 61.6 | 39.6 |  | 50.0 | 27.5 |  | 1.4 | 39.4 | 229.2 | 435.8 |
| 2010 |  | 61.6 | 38.7 |  | 49.0 | 26.8 |  | 1.1 | 39.4 | 221.3 | 417.4 |
| 2011 |  | 60.7 | 37.3 |  | 46.9 | 26.1 |  | 1.2 | 40.6 | 209.0 | 380.1 |
| 2012 |  | 63.5 | 37.9 |  | 47.2 | 27.0 |  | 1.0 | 40.2 | 214.5 | 397.9 |
| 2013 |  | 61.7 | 36.0 |  | 44.8 | 25.7 |  | 1.4 | 38.2 | 202.2 | 369.2 |
| 2014 |  | 61.9 | 35.3 |  | 43.7 | 25.7 |  | 1.2 | 39.6 | 196.8 | 349.5 |
| 2015 |  | 62.6 | 35.1 |  | 41.7 | 27.3 |  | 1.4 | 38.2 | 196.0 | 343.3 |
| 2016 |  | 63.8 | 34.8 |  | 42.6 | 25.8 |  | 1.1 | 41.2 | 191.2 | 333.5 |
| 2017 |  | 64.9 | 34.5 |  | 41.6 | 26.3 |  | 1.4 | 39.0 | 190.3 | 331.5 |
| 2018 |  | 65.4 | 34.0 |  | 40.8 | 26.1 |  | 1.4 | 39.1 | 187.1 | 314.5 |
| 2019 |  | 68.9 | 34.7 |  | 42.0 | 26.5 |  | 1.3 | 39.9 | 191.7 | 324.7 |
| 2020 |  | 64.9 | 32.0 |  | 37.9 | 25.1 |  | 1.5 | 34.1 | 181.1 | 283.1 |
| 2021 |  | 70.3 | 33.2 |  | 38.4 | 27.3 |  | 1.1 | 38.5 | 181.7 | 312.0 |

* Age-standardised incidence using the 2015 world population from the World Bank^1^ as standard population.

**Supp Table 5. Joinpoint regression analysis of the trend in lung cancer incidence between 2002 and 2021**

| **A) Whole population** | | | | | |
| --- | --- | --- | --- | --- | --- |
|  | Measure | Year segment | Estimate (%) | 95% CI | p |
| Crude | AAPC | - | - | - | - |
|  | APC | 2002-2021 | 1.23 | 1.02, 1.44 | <0.001 |
| Age-standardised | AAPC | - | - | - | - |
|  | APC | 2002-2021 | -1.32 | -1.49, -1.15 | <0.001 |
|  |  |  |  |  |  |
| **B) By sex** |  |  |  |  |  |
|  | Measure | Year segment | Estimate (%) | 95% CI | P |
| Male | AAPC | - | - | - | - |
|  | APC | 2002-2021 | -1.92 | -2.12, -1.73 | <0.001 |
| Female | AAPC | - | - | - | - |
|  | APC | 2002-2021 | -0.14 | -0.43, 0.14 | 0.306 |
|  |  |  |  |  |  |
| **C) By age group** | | | | | |
|  | Measure | Year segment | Estimate (%) | 95% CI | p |
| Age <40 | AAPC | - | - | - | - |
|  | APC | 2002-2021 | -0.24 | -1.27, 0.81 | 0.642 |
| Age 40-59 | AAPC | 2002-2021 | 0.72 | -0.02, 1.47 | 0.057 |
|  | APC | 2002-2011 | 2.16 | 0.86, 3.47 | 0.003 |
|  |  | 2011-2021 | -0.55 | -1.55, 0.47 | 0.266 |
| Age 60-79 | AAPC | 2002-2021 | -1.98 | -2.38, -1.59 | <0.001 |
|  | APC | 2002-2015 | -2.36 | -2.73, -1.99 | <0.001 |
|  |  | 2015-2021 | -1.16 | -2.25, -0.05 | 0.042 |
| Age >79 | AAPC | 2002-2021 | -1.94 | -2.72, -1.15 | <0.001 |
|  | APC | 2002-2010 | -0.67 | -2.31, 0.99 | 0.401 |
|  |  | 2010-2021 | -2.85 | -3.70, -1.99 | <0.001 |

AAPC: average annual percent change, APC: annual percent change, CI: confidence interval.

**Supp Table 6. Relative period survival (%) of lung cancer by year period**

| **A) 1-year survival** | | | | | | | | | | | | |
| --- | --- | --- | --- | --- | --- | --- | --- | --- | --- | --- | --- | --- |
| Year period |  | By sex* | |  | By age group | | | |  | By comorbidity severity* | | |
|  |  | Male | Female |  | Age <40 | Age 40-59 | Age 60-79 | Age >79 |  | None | Mild | Severe |
| 2004/2006 |  | 32.6 | 38.4 |  | 40.6 | 44.9 | 34.3 | 15.6 |  | 51.1 | 43.4 | 19.7 |
| 2007/2009 |  | 35.3 | 44.8 |  | 52.7 | 51.1 | 38.4 | 17.7 |  | 54.0 | 47.7 | 25.7 |
| 2010/2012 |  | 38.2 | 52.4 |  | 57.2 | 55.3 | 43.7 | 21.2 |  | 61.2 | 49.5 | 31.3 |
| 2013/2015 |  | 41.0 | 55.6 |  | 64.7 | 60.1 | 46.8 | 23.2 |  | 61.2 | 52.3 | 35.9 |
| 2016/2018 |  | 44.0 | 63.1 |  | 70.9 | 64.1 | 52.7 | 25.6 |  | 65.4 | 59.2 | 40.2 |
| 2019/2021 |  | 49.3 | 67.0 |  | 74.3 | 70.1 | 57.9 | 30.0 |  | 69.6 | 64.2 | 44.3 |
|  |  |  |  |  |  |  |  |  |  |  |  |  |
| **B) 5-year survival** | | | | | | | | | | | | |
| Year period |  | By sex* | |  | By age group | | | |  | By comorbidity severity* | | |
|  |  | Male | Female |  | Age <40 | Age 40-59 | Age 60-79 | Age >79 |  | None | Mild | Severe |
| 2004/2006 |  | 14.0 | 17.0 |  | 18.6 | 19.8 | 14.5 | 6.9 |  | 24.7 | 17.3 | 6.8 |
| 2007/2009 |  | 13.5 | 17.4 |  | 20.5 | 18.2 | 15.4 | 7.0 |  | 24.7 | 19.0 | 6.8 |
| 2010/2012 |  | 14.5 | 21.3 |  | 29.5 | 20.2 | 17.4 | 7.8 |  | 28.8 | 23.5 | 8.0 |
| 2013/2015 |  | 15.7 | 21.4 |  | 20.3 | 23.1 | 18.3 | 7.6 |  | 30.6 | 25.9 | 8.2 |
| 2016/2018 |  | 18.4 | 29.6 |  | 40.7 | 30.0 | 23.3 | 7.5 |  | 36.8 | 31.0 | 11.6 |
| 2019/2021 |  | 22.0 | 35.2 |  | 46.5 | 36.2 | 27.6 | 10.2 |  | 42.2 | 35.7 | 14.1 |

* Age-standardised survival using the world lung cancer population from GLOBOCAN 2020^2^ as standard population.

**Supp Table 7. Annual Percent Change of lung cancer survival between 2004 and 2021**

| **A) By sex** |  |  |  |  |
| --- | --- | --- | --- | --- |
|  | Survival measure | APC (%) | 95% CI | P |
| Male | 1-year | 8.45 | 7.40, 9.51 | <0.001 |
|  | 5-year | 10.45 | 5.06, 16.11 | 0.005 |
| Female | 1-year | 10.85 | 8.16, 13.61 | <0.001 |
|  | 5-year | 17.42 | 10.99, 24.22 | 0.001 |
|  |  |  |  |  |
| **B) By age group** | | | | |
|  | Survival measure | APC (%) | 95% CI | P |
| Age <40 | 1-year | 11.08 | 6.95, 15.36 | 0.002 |
|  | 5-year | 20.79 | 7.19, 36.12 | 0.012 |
| Age 40-59 | 1-year | 8.69 | 7.57, 9.82 | <0.001 |
|  | 5-year | 15.53 | 7.72, 23.92 | 0.005 |
| Age 60-79 | 1-year | 10.81 | 9.82, 11.82 |  |
|  | 5-year | 14.61 | 9.94, 19.47 | 0.001 |
| Age >79 | 1-year | 13.40 | 11.40, 15.44 | <0.001 |
|  | 5-year | 7.21 | 0.23, 14.68 | 0.045 |
|  |  |  |  |  |
| **C) By comorbidity severity** | | | | |
|  | Survival measure | APC (%) | 95% CI | P |
| None | 1-year | 6.10 | 4.34, 7.88 | 0.001 |
|  | 5-year | 12.38 | 8.79, 16.08 | 0.001 |
| Mild | 1-year | 8.22 | 6.53, 9.94 | <0.001 |
|  | 5-year | 15.98 | 14.10, 17.89 | <0.001 |
| Severe | 1-year | 15.50 | 10.92, 20.27 | 0.001 |
|  | 5-year | 17.96 | 9.78, 26.75 | 0.003 |

APC: annual percent change, CI: confidence interval.

**Supp Table 8. Baseline comorbidity severity of the survival cohort**

| Year |  | Total number of incident cases |  | Comorbidity Severity, n (%) | | |
| --- | --- | --- | --- | --- | --- | --- |
|  |  |  |  | None  (CCI score = 0) | Mild  (CCI score 1-2) | Severe  (CCI score ≥ 3) |
| 2002 |  | 3708 |  | 1510 (40.7) | 558 (15.0) | 1640 (44.2) |
| 2003 |  | 3549 |  | 1368 (38.5) | 431 (12.1) | 1750 (49.3) |
| 2004 |  | 3806 |  | 1443 (37.9) | 477 (12.5) | 1886 (49.6) |
| 2005 |  | 3884 |  | 1401 (36.1) | 520 (13.4) | 1963 (50.5) |
| 2006 |  | 3911 |  | 1424 (36.4) | 457 (11.7) | 2030 (51.9) |
| 2007 |  | 3917 |  | 1383 (35.3) | 474 (12.1) | 2060 (52.6) |
| 2008 |  | 3961 |  | 1443 (36.4) | 496 (12.5) | 2022 (51.0) |
| 2009 |  | 4298 |  | 1365 (31.8) | 568 (13.2) | 2365 (55.0) |
| 2010 |  | 4330 |  | 1320 (30.5) | 595 (13.7) | 2415 (55.8) |
| 2011 |  | 4293 |  | 1327 (30.9) | 543 (12.6) | 2423 (56.4) |
| 2012 |  | 4542 |  | 1403 (30.9) | 610 (13.4) | 2529 (55.7) |
| 2013 |  | 4428 |  | 1394 (31.5) | 591 (13.3) | 2443 (55.2) |
| 2014 |  | 4478 |  | 1443 (32.2) | 559 (12.5) | 2476 (55.3) |
| 2015 |  | 4563 |  | 1510 (33.1) | 592 (13.0) | 2461 (53.9) |
| 2016 |  | 4681 |  | 1488 (31.8) | 594 (12.7) | 2599 (55.5) |
| 2017 |  | 4797 |  | 1547 (32.2) | 665 (13.9) | 2585 (53.9) |
| 2018 |  | 4874 |  | 1734 (35.6) | 629 (12.9) | 2511 (51.5) |
| 2019 |  | 5171 |  | 1861 (36.0) | 708 (13.7) | 2602 (50.3) |
| 2020 |  | 4857 |  | 1672 (34.4) | 685 (14.1) | 2500 (51.5) |
| 2021 |  | 5211 |  | 1873 (35.9) | 800 (15.4) | 2538 (48.7) |

Reference:

1. Population estimates and projections: The World Bank Group; [Available from: <https://databank.worldbank.org/source/population-estimates-and-projections>] [Accessed Date: Oct 4, 2023]

2. J F, M E, F L, M C, L M, M P, et al. Global Cancer Observatory: Cancer Today Lyon, France: International Agency for Research on Cancer; 2020 [Available from: <https://gco.iarc.fr/today/home>] [Accessed Date: Oct 4, 2023,
